# Effects of Panthenol and N-Acetylcysteine on Changes in the Redox State of Brain Mitochondria under Oxidative Stress In Vitro

**DOI:** 10.3390/antiox10111699

**Published:** 2021-10-27

**Authors:** Dmitry S. Semenovich, Egor Yu. Plotnikov, Oksana V. Titko, Elena P. Lukiyenko, Nina P. Kanunnikova

**Affiliations:** 1 Institute of Biochemistry of Biologically Active Substances, NAS of Belarus, 230030 Grodno, Belarus; o.titko@mail.ru (O.V.T.); lukgrodno@mail.ru (E.P.L.); n.kanunnikova@grsu.by (N.P.K.); 2 A.N. Belozersky Institute of Physico-Chemical Biology, Moscow State University, 119992 Moscow, Russia; plotnikov@belozersky.msu.ru; 3 Department of Technology, Physiology and Food Hygiene, State University of Grodno, 230030 Grodno, Belarus

**Keywords:** redox state, glutathione system, thiol–disulfide balance, brain mitochondria, oxidative stress, D-panthenol

## Abstract

The glutathione system in the mitochondria of the brain plays an important role in maintaining the redox balance and thiol–disulfide homeostasis, whose violations are the important component of the biochemical shifts in neurodegenerative diseases. Mitochondrial dysfunction is known to be accompanied by the activation of free radical processes, changes in energy metabolism, and is involved in the induction of apoptotic signals. The formation of disulfide bonds is a leading factor in the folding and maintenance of the three-dimensional conformation of many specific proteins that selectively accumulate in brain structures during neurodegenerative pathology. In this study, we estimated brain mitochondria redox status and functioning during induction of oxidative damage in vitro. We have shown that the development of oxidative stress in vitro is accompanied by inhibition of energy metabolism in the brain mitochondria, a shift in the redox potential of the glutathione system to the oxidized side, and activation of S-glutathionylation of proteins. Moreover, we studied the effects of pantothenic acid derivatives—precursors of coenzyme A (CoA), primarily D-panthenol, that exhibit high neuroprotective activity in experimental models of neurodegeneration. Panthenol contributes to the significant restoration of the activity of enzymes of mitochondrial energy metabolism, normalization of the redox potential of the glutathione system, and a decrease in the level of S-glutathionylated proteins in brain mitochondria. The addition of succinate and glutathione precursor N-acetylcysteine enhances the protective effects of the drug.

## 1. Introduction

An increase in the average life expectancy of people in modern society contributes to changes in the structure of morbidity and leads to a rise in the number of degenerative diseases, which are based on the mechanisms of cellular apoptosis. Neurodegenerative diseases cause significant brain dysfunction and lead to a sharp decrease in the quality of life of elderly patients [1]. The participation of free radicals and oxidative stress in the development of various neurodegenerative diseases is widely described in the literature [2,3,4]. Still, there is evidence that reactive oxygen species (ROS) are the primary cause of neuronal death; on the other hand, ROS are generated in response to the activation of certain signaling cascades, as was shown for tumor necrosis factor α-induced cell death or programmed necrotic cell death [5,6,7,8,9].

Activation of lipoperoxidation is one of the most striking manifestations of brain damage in neurodegenerative pathology [2,3,8]. The presence of a high level of malonic dialdehyde or other thiobarbituric acid-reactive substances (TBARS) in the fraction of synaptosomal membranes in the brain has been demonstrated for Alzheimer’s disease (AD) and some other neuropathologies [10]. It is known that the induction of oxidative stress in AD is similar to that in acute brain pathologies (traumatic or ischemic). However, the level of free radicals in AD is not so high as to lead to a failure of the antioxidant defense system and cause apoptotic death of neurons [11,12].

Age, inflammation, the influence of environmental factors, and some nutritional factors can also contribute to a decrease in antioxidant defense systems and an increase in the formation of free radical oxidation products [13,14,15]. However, in genetic experiments, it was found that activation of oxidative stress by deleting Superoxide Dismutase (SOD) genes did not decrease the lifespan in fruit flies, even when all SOD genes were deleted [6]. Under certain conditions, increased levels of free radicals can even lengthen life. Based on this, it was suggested that although oxidative stress is activated with age, it is not the cause of aging [6].

Modern concepts of the role of oxidative stress in the development of Parkinson’s disease (PD), AD, and other neurodegenerative diseases indicate that the mechanisms of initiation and progress of neurodegenerative disorders include not only oxidative stress and changes in the activity of key enzymes for scavenging active radicals, but also disruption of thiol homeostasis and signaling pathways, alteration in protein conformation, and its aggregation [16,17,18].

In recent years, an increasing amount of evidence linked mitochondrial dysfunction with neurodegenerative diseases [19,20,21,22,23]. Mitochondria are considered to be the main site for the formation of excess free radicals, disturbances in energy metabolism, and a shift in the thiol–disulfide balance in cells. The effect of mitochondrial damage on the processes of cell destruction is most pronounced in neurons because mitochondria play an essential role in the initiation of apoptosis-programmed cell death [4,24,25]. Mitochondrial glutathione may play an important role in regulating apoptosis [8,26,27,28]. Reduced glutathione (GSH) is a vital component in all cells, including neuronal ones, participating in the maintenance of redox status and being a cofactor of signal transduction and the antioxidant defense system, especially in the brain [26,28]. A shift of the glutathione redox ratio (GSH/GSSG) is vital for cell function. GSH is a substrate for enzymes that scavenge free radicals, inactivate electrophilic groups, and maintain reduced cysteine-thiol groups in proteins by appropriate GSSG formation. Changes in GSH content and the activity of GSH-dependent enzymes cause the development of mitochondrial dysfunction, accumulation of ROS and reactive nitrogen species (RNS), impaired signaling pathways, protein aggregation, cell damage, and, ultimately, death [2,29,30,31]. Maintaining the redox balance is important not only to prevent damage caused by free radical oxidation products but also to maintain proper redox signaling through ROS and RNS as secondary messengers [16,17]. The primary function of the redox buffer systems in cells is to protect thiol groups from oxidation and to restore those that have been oxidized due to normal or altered cell metabolism.

Disturbances in protein folding and the formation of protein aggregates are typical features of neurodegenerative diseases [32,33]. The formation of disulfide bonds is a critical moment in folding and maintaining the three-dimensional conformation of many proteins [34]. In turn, specific proteins produced in certain types of neurodegenerative pathologies can affect mitochondrial function. Modulation of protein functions through S-glutathionylation and/or S-nitrosylation of proteins affects many cell functions, ranging from resistance to oxidative stress, post-translational protein modification, and up to transcriptional activation and inhibition of proteasomes. The central role in these oxidative modifications is played by glutathione, whose availability and redox status seems to determine the rate of oxidative modification of proteins [35,36].

The new knowledge accumulated to date about the structure and functions of the brain could be affected by metabolic alterations in brain cells and subcellular compartments. Eventually, it led to the intensive search for antioxidative and metabolic-related approaches to treating neurodegenerative pathology. Given the gradual development and long-term course of neurodegenerative diseases, the study of the effect of metabolic therapies which are non-toxic and allow us to support the processes of energy formation and maintenance of redox balance in the cell is becoming more and more urgent.

In this study, we carried out in vitro experiments on isolated mitochondria of the cerebral hemispheres of rats upon initiation of oxidative stress to elucidate the relationship between the formation of ROS-related products, energy metabolism, changes in the redox potential of the glutathione system, and post-translational S-glutathionylation of proteins during the oxidative challenge. We use tert-butyl hydroperoxide (tBHP) or iron sulfate to induce oxidative stress in mitochondria. tBHP is an analog of endogenous membrane lipid hydroperoxides, known to cause the formation of Schiff bases, malondialdehyde, 4-hydroxynonenal, and protein carbonyls. Fe^2+^ ions ignite lipid peroxidation through Fenton’s reaction, which leads to oxidation of the cell’s low molecular weight compounds, lipids, and proteins. Panthenol, succinate, and the precursor of glutathione biosynthesis N-acetylcysteine (NAC) were used as modulators of the redox balance.

## 2. Materials and Methods

### 2.1. Materials

Chemicals were obtained from the following sources: Tris base, Percoll, tert-butyl hydroperoxide, manganese chloride, cis-aconitic acid, iron(II) sulfate heptahydrate, L-citric acid, isocitric acid, triethanolamine-HCl, Triton X-100, sucrose, 2-vinylpyridine, β-nicotinamide adenine dinucleotide phosphate reduced (β-NADPH), glutathione reductase from baker’s yeast, D-panthenol, N-acetylcysteine, sodium 2-oxoglutarate, sodium succinate, sodium azide, 2,3-naphthalenedicarboxaldehyde, NP-40, N-Ethylmaleimide (NEM), Tris(2-carboxyethyl)phosphine hydrochloride (TCEP), 3-[4,5-dimethylthiazol-2-yl]-2,5-diphenyltetrazolium bromide (MTT), phenazine methosulfate (PMS), isocitrate dehydrogenase, and purified porcine aconitase (30 units/g) from Sigma-Aldrich (Merck); 2-thiobarbituric acid, reduced and oxidized glutathione, 5,5′-Dithio-bis-(2-nitrobenzoic Acid) (DTNB), trichloroacetic acid (TCA), Coomassie Brilliant Blue G250 from AppliChem. All reagents were of analytical grade, HPLC grade, or the best available pharmaceutical grade.

### 2.2. Animals

The experiments were carried out on mitochondria isolated from the tissue of the cerebral hemispheres of intact male Wistar rats weighing 180–200 g (n = 16), kept under standard conditions of the animal facilities. The animals had unlimited access to food and water and were kept in cages in a temperature-controlled environment (20 ± 2 °C) under the 12/12 h light/dark regime. The experiments were conducted in accordance with the ethical standards and recommendations for accommodation and care of laboratory animals, covered by the Council Directives of the European community 2010/63/EU on the use of animals for experimental studies. The experiments were approved by the Ethics Committee of the Institute of Biochemistry of Biologically Active Substances (Protocol 4/19 from 1 April 2019). Animals were humanely euthanized in accordance with accepted bioethical requirements. The brain was removed, immersed in a cold isolation medium, and used to isolate mitochondria as described below.

### 2.3. Isolation of Brain Mitochondria

Mitochondria were isolated by differential centrifugation in a Percoll gradient as described earlier [37]. The cerebral hemispheres were quickly removed and were homogenized (1:9, *w*/*v*) in a glass Teflon homogenizer with ice-cold isolation medium containing 320 mM sucrose, 10 mM Tris-HCl, and 1 mM EDTA, pH 7.4 (at 4 °C). The homogenate was centrifuged at 1300× *g* for 5 min, and the supernatant was centrifuged at 21,000× *g* for 10 min (at 4 °C). The obtained crude mitochondrial pellet was washed in cold isolation medium and was dissolved in 15% Percoll. Mitochondria were purified in a density gradient of 15, 23, and 40% Percoll (in a volume ratio of 2.3:2.5:1, respectively) by centrifugation for 8 min at 31,000× *g* (at 4 °C).

The mitochondrial band was collected and washed twice with a buffer containing 125 M KCl, 10 mM Tris-HCl, pH 7.4, and resuspended in this buffer to a total protein concentration of 1.5–2.0 mg/mL. Total protein concentration was determined by the Bradford method [38].

### 2.4. Modeling Oxidative Stress in Mitochondria and Adding Metabolic Protectors In Vitro

Oxidative stress was induced by adding tert-butyl hydroperoxide (tBHP) or iron(II) sulfate (FeSO_4_) to the mitochondrial suspension. In experiments with tBHP, mitochondria were incubated at 37 °C for 15 min after the addition of 0.1 or 0.6 mM tBHP. In other experiments, oxidative stress was induced by incubating mitochondria with 0.05 or 0.1 mM FeSO_4_ for 45 min at 37 °C. Before the induction of oxidative stress, mitochondria were preincubated for 30 min (37 °C) with various concentrations (0.01–1 mM) of metabolic protectors–D-panthenol (PL), N-acetylcysteine (NAC) and sodium succinate (5 mM). After the incubation was completed, the samples were placed on ice, and aliquots were taken for further biochemical studies. The concentrations of used compounds were chosen based on the previous studies.

### 2.5. Free and Protein-Bound TBARS Assay

The products of lipid peroxidation were assessed by the level of 2-thiobarbituric acid reactive compounds (TBARS) [39]. An equal volume of cold 10% TCA was added to the suspension of mitochondria and centrifuged for 10 min at 6000 rpm (at 4 °C).

Free TBARS was determined by mixing 500 μL of the supernatant with 100 μL of TBA reagent (1.25% TBA, 41.5% TCA, 1 M HCl), 6 μL of 0.1 M butylated hydroxytoluene and boiling in a water bath for 20 min. The samples were cooled in an ice bath, 600 μL of 1-butanol was added, shaken, and centrifuged at 6000 rpm for 10 min. The absorbance of the butanol extract was measured at 532 nm.

Alkaline hydrolysis was carried out to determine protein-bound TBARS. The protein precipitate was dissolved in 500 μL of 0.1 M sodium hydroxide and incubated for 30 min at 60 °C. Then the mixture was cooled in an ice-water bath, clear protein solution was collected after centrifugation (at 6000 rpm for 10 min) and TBARS was measured as described above. The TBARS content (nmol/mg protein) was expressed using the millimolar extinction coefficient 156 mM^−1^·cm^−1^.

### 2.6. Assay of TCA Enzymes Activity

The activity of aconitase was assayed by determining the rate of formation of the intermediate product, cis-aconitate, from the L-citrate [40]. The enzymatic reaction was initiated by adding mitochondrial suspension to the reactive mixture containing 20 mM triethanolamine-HCl (pH 7.5), 10 mM cysteine-HCl, and 1.0 mM trisodium L-citrate. The changes of absorbance at 240 nm were recorded for 5 min. The millimolar extinction coefficient of cis-aconitate at 240 nm is 4.88 mM^−1^·cm^−1^.

Activities of succinate dehydrogenase (SDH) and 2-oxoglutarate dehydrogenase (OGDH) were assessed by using potassium ferricyanide as an electron acceptor as described in [41,42]. The activity of 2-oxoglutarate dehydrogenase was determined in a reaction medium containing 50 mM potassium phosphate buffer, pH 7.4, 1 mM MgCl_2_, 3 mM sodium 2-oxoglutarate, 0.2 mM thiamine diphosphate, 0.6 mM potassium ferricyanide. The succinate dehydrogenase activity was determined in a reaction medium containing 100 mM potassium phosphate buffer, pH 7.4, 10 mM sodium succinate, 2.5 mM EDTA, 15 mM sodium azide, and 2 mM potassium ferricyanide. The reaction was started by adding a suspension of mitochondria to the reaction medium and stopped after 30 min by adding cold 10% TCA. The samples were centrifuged for 5 min at 6000 rpm. In the supernatant, the change in the content of ferricyanide was determined spectrophotometrically at a wavelength of 420 nm using a millimolar extinction coefficient of 1.02 mM^−1^·cm^−1^.

### 2.7. Determination of the Thiol Redox State

To determine lipid peroxidation parameters, level of protein and non-protein thiols, and the glutathione system, 10% TCA was added to the samples. They were centrifuged for 10 min at 6000 rpm at 4 °C. The supernatant was assayed for the contents of reduced (GSH) and oxidized (GSSG) glutathione. The level of protein thiols and S-glutathionylated proteins (PSSG) were determined in the protein precipitate.

The content of GSH and oxidized GSSG was measured by the cyclic enzymatic method with glutathione reductase [43]. To determine GSH, triethanolamine was added to 100 μL of the supernatant to pH 7. To determine GSSG, 2 μL of 2-vinylpyridine was added to 100 μL of the supernatant and incubated for 1 h at room temperature, the pH was neutralized to 7 with triethanolamine. Then a reaction mixture containing 70 mM potassium phosphate buffer, pH 7.5, 3.5 mM EDTA, 0.4 IU glutathione reductase from baker’s yeast, 0.2 mM DTNB, and 0.05 mM NADPH was added to the samples to a final volume of 1 mL. The increase in absorption was recorded at 412 nm for 5 min at a temperature of 37 °C. A calibration curve was constructed using oxidized glutathione (0.5–2 nmol).

The level of protein thiols was measured with Ellman’s reagent [44]. The protein precipitate was solubilized in 0.43 M Tris-HCl buffer, pH 8.6, containing 10.2 M urea, 0.46 M glycine, 2.7 mM EDTA. Next, 5 mM DTNB was added to the samples and incubated at room temperature for 30 min. The absorbance at 412 nm was determined and the content of protein thiols was expressed using a millimolar extinction coefficient of 13.6 mM^−1^·cm^−1^.

The level of S-glutathionylated proteins was measured by the spectrofluorimetric method using 2,3-naphthalenedicarboxaldehyde [45]. The protein pellet was solubilized in 20 mM Tris-HCl buffer, pH 8.0, containing 1% NP-40, 2 mM EDTA, and 2 mM NEM. Excess NEM was removed by extraction with cold acetone (−20 °C). Disulfide bonds in the protein were reduced with 5 mM TCEP in a medium containing 0.5 M Tris-HCl pH 8.0, 0.1% Triton X-100. Glutathione released as a result of the reduction of disulfide groups of the protein was determined by reaction with 2,3-naphthalene carboxaldehyde. The sample was mixed with 10 mM 2,3-naphthalenecarboxaldehyde in DMSO at a ratio of 1:9 (*v*/*v*) and incubated in the dark at room temperature for 30 min. Fluorescence was determined with an excitation wavelength of 485 nm and an emission wavelength of 520 nm. The results were calculated as nmol GSH/mg protein to a standard curve generated with GSH.

### 2.8. Statistical Analysis

The experimental data were statistically processed using Microsoft Excel 2016 and GraphPad Prism 6.0. The experimental data are presented as mean ± SEM. The significance of differences was assessed using a one-way analysis of variance (ANOVA) using Tukey’s test. In all cases, the differences were considered statistically significant at *p* < 0.05.

## 3. Results

### Protective Effect of PL in tBHP-Induced Oxidative Stress of Mitochondria In Vitro

The study of the effect of tBHP on mitochondria from the rat brain showed that 30-min incubation with tBHP at 37 °C led to a three-fold increase in the level of free TBARS (*p* < 0.05), while the content of protein-bound TBARS rose to 36.8% (*p* < 0.05) in mitochondria (Table 1), which is an index of the activation of lipid peroxidation. To study the effect of CoA precursors, panthenol (PL) with final concentrations of 10, 25, 50, and 100 μM was added to the mitochondrial suspension in samples and incubated for 30 min at 37 °C before adding tBHP to the incubation medium. We have shown that panthenol in a dose-dependent manner contributed to a decrease in both parameters, which, however, did not reach the control level even in the presence of 100 μM panthenol. It should be noted that in our experiments, PL (0.01–1 mM) alone does not cause changes in the TBARS level in mitochondria.

In the presence of tBHP (600 μM) in the incubation medium of mitochondria, there was a decrease in succinate dehydrogenase activity by 29% (*p* < 0.05), OGDH activity by 25% (*p* < 0.05), and aconitase activity by 30% (*p* < 0.05) (Table 2). The addition of panthenol (50–100 μM) significantly contributed to the restoration of the activity of all the above enzymes close to the values in the control group.

The study of the content of the thiol in the brain mitochondria showed that in the presence of tBHP levels of protein thiols dropped by 10% (Table 3). The addition of PL increased these parameters in a dose-dependent manner, contributing to the restoration of thiol levels to near-control values.

**Figure 1:**
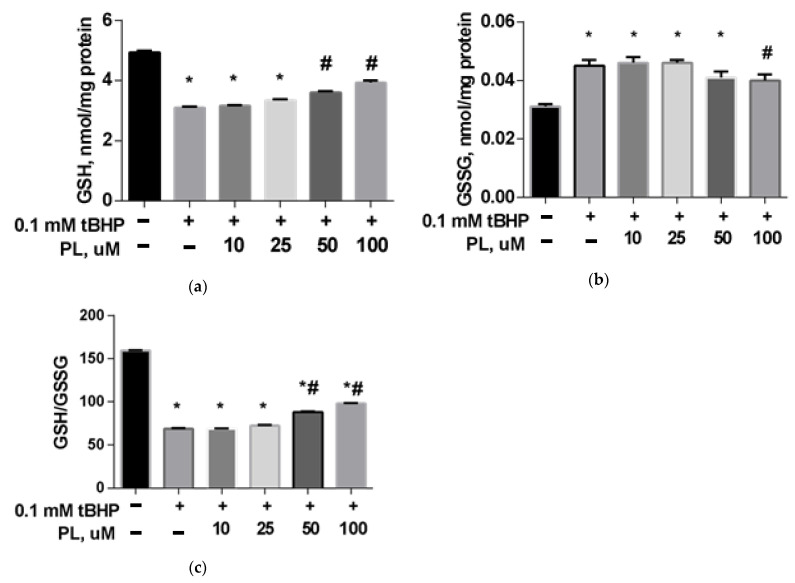
The level of GSH (**a**), GSSG (**b**), and GSH/GSSG ratio (**c**) in the brain mitochondria after treatment with tBHP (0.1 mM) and the effect of different concentrations of PL. Note: * *p* < 0.05 compared to control, # *p* < 0.05 compared to tBHP.

The GSH content in the brain mitochondria in the tBHP-treated group decreased by 31% (Figure 1a) compared to the control (*p* < 0.05), the GSSG content increased 1.5 times (Figure 1b), and the GSH/GSSG ratio decreased more than two times (Figure 1c), which reflects a significant decrease in the reducing capacity of the glutathione system. The addition of PL (50–100 μM) provided the return of these parameters closer to the values found in the control group (Figure 1).

**Figure 2:**
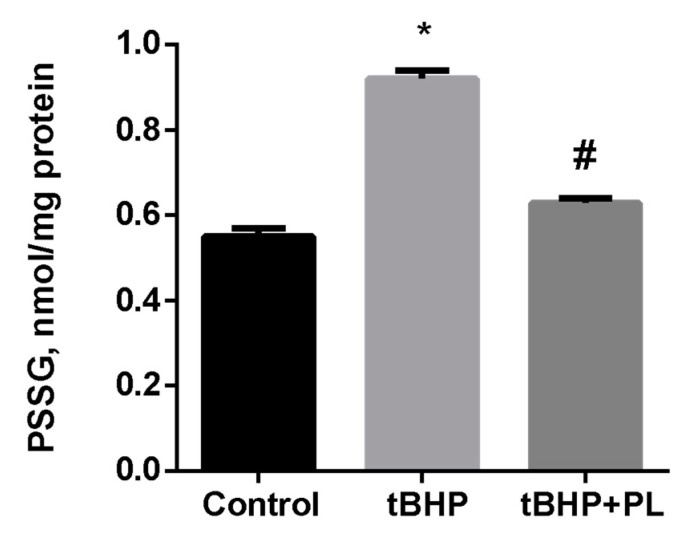
The level of PSSG (nmol GSH/mg protein) in the mitochondria treated with tBHP (0.1 mM) and effects of 0.1 mM of PL. Note: * *p* < 0.05 compared to control, # *p* < 0.05 compared to tBHP.

Pronounced changes were observed concerning the level of S-glutathionylated proteins (PSSG) in tBHP-treated mitochondria. Tert-butyl hydroperoxide increased the PSSG level by 67% (*p* < 0.05), while the addition of 100 μM PL reduced the PSSG level to almost control values (Figure 2).

Our results confirm a correlation between the activation of free radicals production, disturbances in energy metabolism, and shifts in the thiol–disulfide balance at the level of subcellular fractions. Thus, in the brain mitochondria, an increase in the content of lipid peroxidation end-products is accompanied by a decrease in the activity of enzymes of the Krebs cycle, as well as a reduction in the content of both high molecular weight protein thiols and low molecular weight thiols (a decrease in the GSH level). The most pronounced changes under oxidative stress conditions were observed with the GSH/GSSG ratio, which fell by more than two times, and the PSSG content, which increased by 67%. Thus, shifts in the GSH/GSSG ratio to oxidated state and increase in S-glutathionylated proteins levels are sensitive indices of changes in the redox balance of the glutathione system and post-translational modification of proteins in brain mitochondria under oxidative stress. Under these conditions, panthenol reduces the shifts in the redox potential of the glutathione system; S-glutathionylation of proteins, however, does not bring them to the control values.

**Figure 3:**
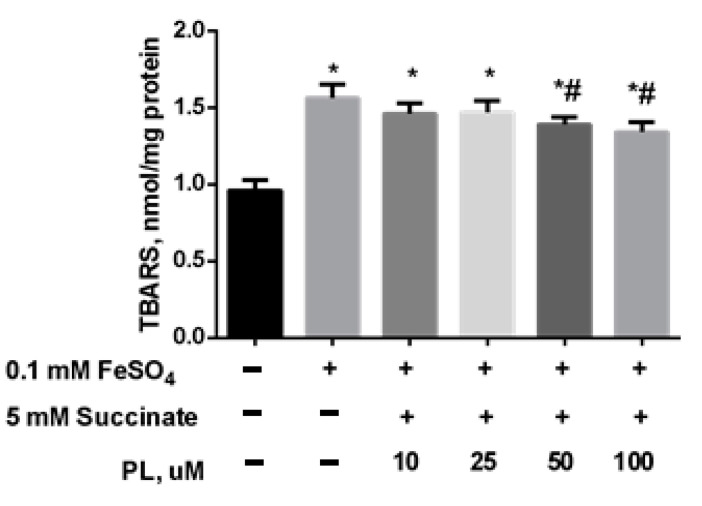
The effect of FeSO_4_ (0.1 mM) on the levels of TBARS (nmol/mg protein) in the brain mitochondria and protective effects of different concentrations of PL. Note: * *p* < 0.05 compared to control, # *p* < 0.05 compared to FeSO_4_.

In the following series of experiments, we initiated the oxidative stress by adding 0.05 mM FeSO_4_ to the mitochondrial incubation medium. It was found that the presence of iron sulfate in the incubation medium also provoked an increase in lipid peroxidation: the content of total TBARS increased by 61% (Figure 3) while PL dose-dependently decreased this parameter but did not bring it to the control level, even in the presence of 100 μM of the substance (Figure 3).

**Figure 4:**
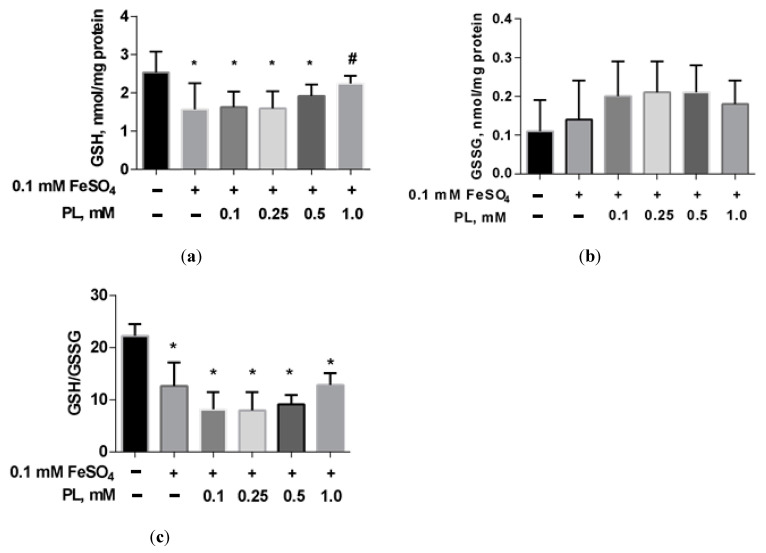
The level of GSH (**a**), GSSG (**b**), and GSH/GSSG ratio (**c**) in brain mitochondria after FeSO_4_ treatment (0.1 mM) and the effect of different concentrations of PL. Note: * *p* < 0.05 compared to control, # *p* < 0.05 compared to FeSO_4_.

In the presence of FeSO_4_, a 38% decrease in GSH and a 43% GSH/GSSG ratio were shown (Figure 4). The addition of panthenol to the incubation medium dose-dependently reduced these deviations.

**Figure 5:**
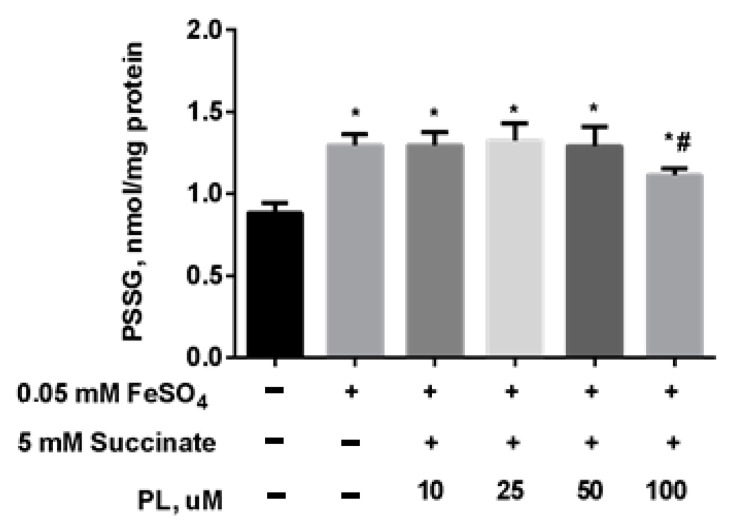
The content of PSSG (nmol/mg protein) in the brain mitochondria after incubation with FeSO_4_ (0.05 mM), succinate (5 mM) and various concentrations of PL. Note: * *p* < 0.05 compared to control, # *p* < 0.05 compared to FeSO_4_.

At the same time, the content of S-glutathionylated proteins in mitochondria increased 1.5 times against the background of oxidative stress (Figure 5), and in the presence of 100 μM panthenol, there was a tendency for it to return to control values.

**Figure 6:**
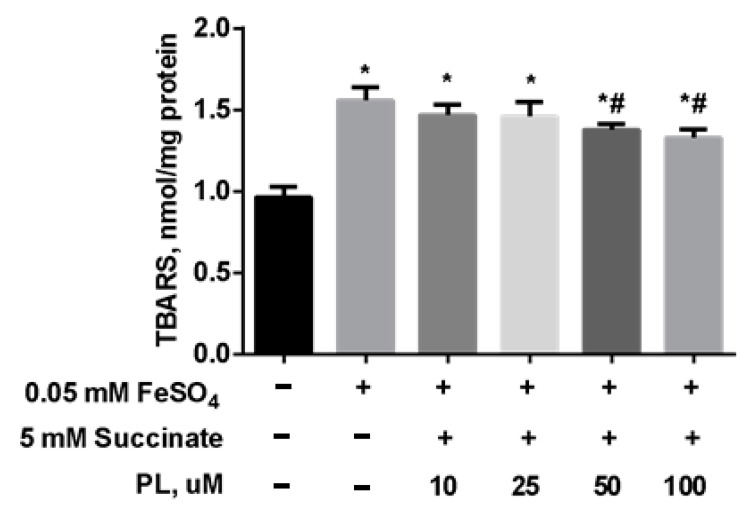
The level of TBARS (nmol/mg protein) in the brain mitochondria after incubation with FeSO_4_ (0.05 mM), succinate (5 mM) and different concentrations of PL. Note: * *p* < 0.05 compared to control, # *p* < 0.05 compared to FeSO_4_.

To test the possibility of enhancing the neuroprotective effect of panthenol, we studied the effect of the combination of PL with succinate on the redox balance in the mitochondria of the brain against the background of oxidative stress initiated by the addition of FeSO_4_. It was found that the addition of FeSO_4_ to the incubation medium was accompanied by an increase in the TBARS content by 67% in mitochondria (Figure 6). In the presence of 0.1–1 mM PL + 5 mM succinate, this effect was weakened in a dose-dependent manner, but the TBARS level did not return to the values in control.

The combination of panthenol + succinate caused less pronounced changes in the redox potential of the glutathione system; however, it did not reach the values in the control group (Table 4).

**Figure 7:**
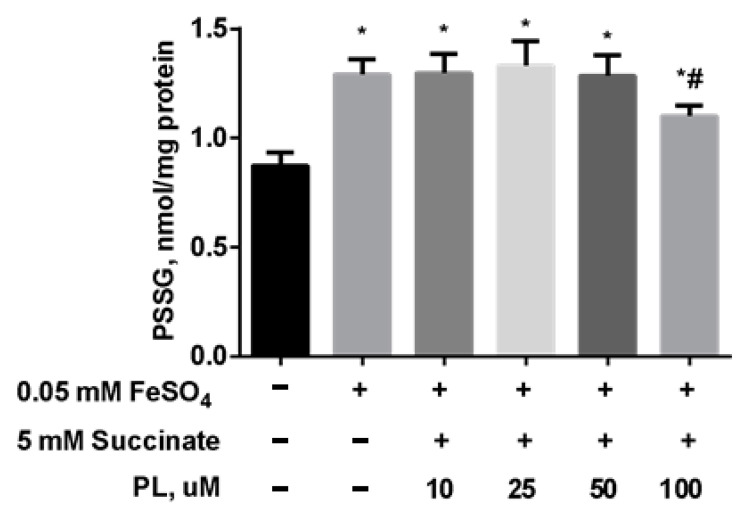
The level of PSSG (nmol GSH/mg protein) in brain mitochondria after the incubation with FeSO_4_ (0.05 mM), succinate (5 mM), and different concentrations of PL. Note: * *p* < 0.05 compared to control, # *p* < 0.05 compared to FeSO_4_.

As in the previous experiment, the content of S-glutathionylated proteins significantly increased after incubation with FeSO_4_ (Figure 7), while the combination of PL + succinate caused only a slight weakening of this effect.

**Figure 8:**
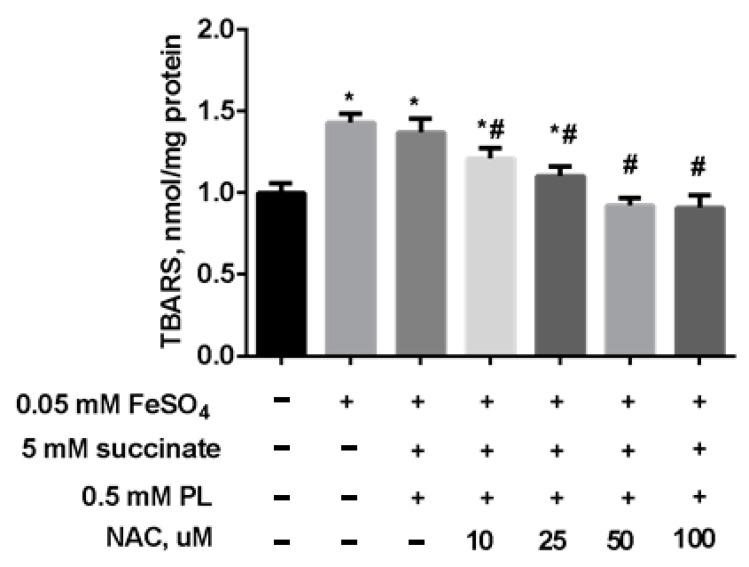
The level of TBARS (nmol/mg protein) in brain mitochondria after the incubation with FeSO_4_ (0.05 mM), succinate (5 mM), panthenol (0.5 mM) and different concentrations of NAC. Note: * *p* < 0.05 compared to control, # *p* < 0.05 compared to FeSO_4_.

In the following experiment, we preincubated mitochondria for 15 min (37 °C) in the presence of 5 mM succinate, 0.5 mM panthenol, and different concentrations of NAC. Thereafter, to induce oxidative stress, a solution of 0.05 mM FeSO_4_ was added to the suspension, and the mixture was incubated at 37 °C for 45 min. The addition of NAC to the combination of succinate and panthenol increased the normalizing effect of the drugs: as the concentration of NAC increased, the level of TBARS decreased up to its normalization in the presence of 0.25 mM NAC and even to values less than in the control group, in the presence of 0.5–1 mM NAC (Figure 8).

The changes in the redox potential of the glutathione system caused by the action of FeSO_4_ in the presence of small doses of NAC + panthenol + succinate decreased, and the GSH level and GSH/GSSG ratio returned to control values only in the presence of the combination of panthenol + succinate + 1 mM NAC (Table 5).

**Figure 9:**
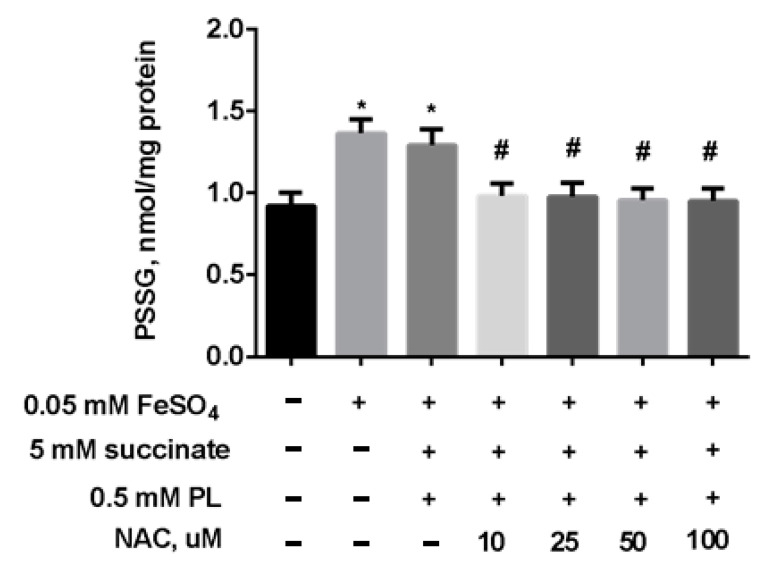
The level of PSSG (nmol GSH/mg protein) in brain mitochondria during the incubation with FeSO_4_ (0.05 mM), succinate (5 mM), PL (0.5 mM), and different concentrations of NAC. Note: * *p* < 0.05 compared to control, # *p* < 0.05 compared to FeSO_4_.

The same efficacy of the protection was demonstrated for the combination of panthenol + succinate + NAC against the increase in S-glutathionylated proteins levels in mitochondria (Figure 9). We observed an almost complete return of this parameter to control values when FeSO_4_ acts in the presence of panthenol, succinate, or 10–100 μM NAC.

## 4. Discussion

Mitochondria are an excellent example of subcellular organelles whose function is closely related to maintaining redox balance. They are the main intracellular oxygen consumption site and the primary source of ROS, most of which is produced by the respiratory chain. Typical signs of metabolic damage in brain tissue under neurodegenerative pathologies are oxidative stress, disturbances in bioenergetics, and a shift in the redox balance [2,3,8,9,13]. Our study confirms that the effect of pro-oxidants on lipoperoxidation processes and energy metabolism is observed not only when acting at the level of the whole organism, organs, and tissues, but also when they act at the level of mitochondria. Incubation of mitochondria with tBHP or FeSO_4_ led to an increase in the content of lipid peroxidation end-products and a decrease in the activity of enzymes of the Krebs cycle.

Even more pronounced changes were observed in the parameters of the glutathione system and S-glutathionylation of the protein, when, following oxidative stress, the level of GSH decreased, and the GSH/GSSG ratio sharply dropped, but the levels of S-glutathionylated proteins simultaneously rose up. This indicates that an important role in maintaining the redox balance in mitochondria is played by the glutathione system and oxidative stress shift. Alterations in GSH and GSSG levels led to a shift in the redox potential of the glutathione system to an oxidized state. Apparently, the change in the redox balance can be associated with a weakening of the activity of the tricarboxylic acid cycle and the simultaneous activation of the pentose phosphate pathway and, accordingly, an increase in the formation of NADPH [46,47]. A decrease in the activity of the Krebs cycle’s enzymes is one of the impacts of oxidative stress effect on the development of neurodegenerative pathologies [48,49]. This effect could be associated with the post-translational modification of succinate dehydrogenase and 2-oxoglutarate dehydrogenase due to glutathionylation [50,51,52,53]. S-glutathionylation of a protein is a specific post-translational modification resulting from the disulfide adduction of GSH with reactive cysteine on the target protein. This glutathionylated disulfide can protect proteins from irreversible oxidation of cysteine [53,54,55]. In addition to the protection of proteins, S-glutathionylation plays the role in the mechanism of redox signaling and dynamic regulation of protein function [56,57]. The GSH-dependent antioxidant system can also participate in the modulation of protein degradation pathways involved in the regulation of cell survival [12].

Recently, much attention has been paid to the search for redox-modulating pharmacological agents for the prevention of oncological, endocrinological, and cardiovascular pathologies associated with oxidative stress, as well as for the prevention and correction of neurodegeneration [31,57].

The brain’s ability to restore functional activity after the ignition of the apoptotic process primarily depends on the depth of energy metabolism impairment in the neural cells [48,49,53]. The most effective methods of normalizing energy metabolism are the restoration of the NAD-dependent site of the tricarboxylic acid cycle, as well as the stimulation of an alternative pathway, which is the oxidation of succinic acid, bypassing the components of the electron-transport chain that are inactive due to a decrease in oxygen content. The intensity of oxidation of the exogenous succinate increases sharply under increased oxygen consumption, and derivatives of succinic acid are supposed as effective antioxidants [58,59]. Treatment with succinic acid increases microcirculation in the brain without affecting blood pressure and heart function [60].

Pantothenic acid derivatives are precursors of coenzyme A (CoA) and effective modulators of the thiol–disulfide system, stimulating an increase in the antioxidant capacity of the nervous tissue by stabilizing the glutathione system, attenuation of lipid peroxidation, and free radical oxidation processes during neurotoxicity in vivo [61,62]. Even though the concentration of CoA and glutathione in the nervous tissue differs significantly: 0.06–0.1 mM of CoA against 1–3 mM of GSH, it was found that administration of pantothenic acid derivatives stabilizes the glutathione system under conditions of oxidative stress [63,64]. The effect of CoA biosynthesis modulators is also observed in the activity of dehydrogenases of the pentose phosphate pathway, indicating the activation of the compensatory pathway of NADPH production when glutathione pool and the tricarboxylic acid cycle are affected in the central nervous system [64].

**Figure 10:**
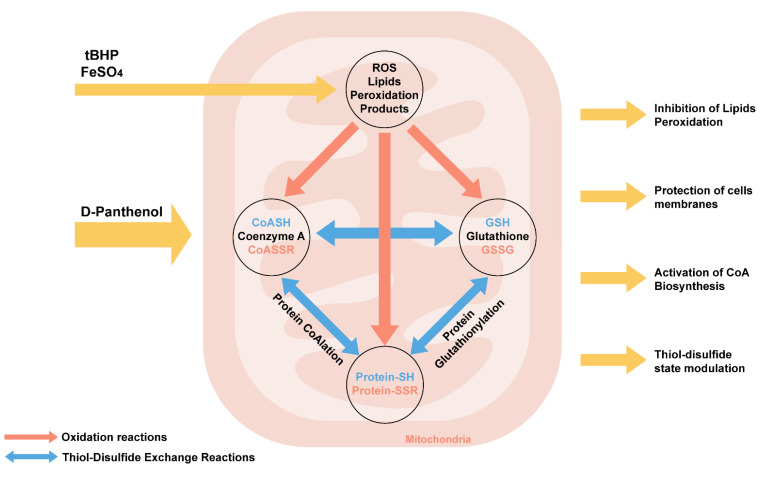
Biochemical mechanisms of effects of D-panthenol on the initiation of oxidative stress in mitochondria in vitro.

We have summarized the biochemical effects of panthenol action during oxidative stress (Figure 10).

PL reduces lipid peroxidation during oxidative stress caused by tBHP and FeSO_4_ in mitochondria in vitro. PL is a lipophilic compound, exerting a stabilizing effect on membrane phospholipids. Probably, due to the alcohol hydroxyl group, PL can exhibit radical scavenger activity, being oxidized to pantothenate. The oxidation of PL to pantothenate in a reaction catalyzed by alcohol dehydrogenase in vivo promotes its inclusion in the biosynthesis of coenzyme A (CoASH). Coenzyme A (CoASH) is predominantly compartmentalized in mitochondria. Along with glutathione, the concentration of CoA in the mitochondrial matrix can reach up to 5 mM. CoASH and its mixed disulfide with glutathione undergo thiol–disulfide exchange reactions with glutathione (GSH) and glutathione disulfide (GSSG) in vitro.

Oxidized forms of CoA and glutathione promote the initiation of covalent modification of proteins-CoAlation and glutathionylation, which actively occur during oxidative and metabolic stress, which is accompanied by a change in the activity of TCA enzymes (in particular, aconitase, 2-oxoglutarate dehydrogenase, etc.) and a decrease in NADH production [64]. Earlier in our works, it was shown that modulation of the biosynthesis of coenzyme A due to the introduction of PL in animals against the background of the neurotoxic action of aluminum chloride leads not only to an increase in free CoA but also glutathione, while a decrease in the content of glutathionylated proteins and lipid peroxidation products in the structures were noted in rat brain [65].

While succinate can maintain energy metabolism in various pathological situations, panthenol enhances its protective effects against redox impairment in the brain mitochondria in an experimental model of cerebral ischemia-reperfusion [66].

It is known that the NAC, the precursor of the biosynthesis of glutathione, improves mitochondrial respiration and reduces the manifestations of oxidative stress in experimental models of neurodegeneration [65,66,67,68,69,70]. We found the addition of NAC to panthenol and succinate treatment increases their protective effects up to complete normalization of lipid peroxidation indicators, the redox potential of the glutathione system, and S-glutathionylation of proteins caused by oxidative stress in the brain mitochondria. Note that because NAC was not removed from the incubation medium when oxidative stress was induced, it could interfere with the surrounding environment of the mitochondrion and have some additional effects apart from those described above. Nevertheless, the protective effects detected on isolated mitochondria are also confirmed by the effects in vivo, in the neuroprotective effect of pantothenic acid derivatives (panthenol, pantethine, and homopantothenic acid) in experimental models of neurodegeneration in rats [66,71]. The use of a combination of panthenol, succinate, and NAC in restoring the disturbed redox balance in the rat brain in experimental models of aluminum neurotoxicity was also successful [72].

Therefore, changes in the redox potential of the glutathione system and S-glutathionylation of proteins are sensitive indicators of oxidative-mediated damage in the brain tissue and the risk of neurodegeneration. The study of the redox-modulating ability of substances for metabolic therapies will expand the opportunities for their use as supporting therapeutic agents for the long-term treatment of neurodegenerative diseases.

## 5. Conclusions

The initiation of oxidative stress in brain mitochondria by tBHP or FeSO_4_ treatment resulted in inhibition of energy metabolism, a shift in the redox potential of glutathione to the oxidized side, and an increase in the content of S-glutathionylated proteins.

Panthenol promoted inhibition of lipid peroxidation, improved energy metabolism, restored the redox potential of the glutathione system, and decreased the levels of S-glutathionylated proteins. The succinate enhanced the protective effects of the panthenol. The most pronounced protective effect against lipoperoxidation and the disturbances in the glutathione system was shown when mitochondria were incubated with NAC along with panthenol. The combination of NAC with panthenol and succinate reduced the production of ROS and restored the redox state of the glutathione system and the levels of S-glutathionylated proteins to the control values. The enhancement of the protective effects of panthenol upon the addition of succinate and N-acetylcysteine indicates the promising use of combinations of these compounds as regulators of the redox status and energy metabolism in brain tissue under oxidative stress conditions inherent for the development of brain degeneration processes. Maintaining the intracellular redox balance can be an important tool for minimizing neurons’ damage and a promising direction in searching for new approaches of pathogenic therapy of neurodegenerative diseases.

## Figures and Tables

**Table 1:** The level of TBARS (nmol/mg protein) in the brain mitochondria after treatment with tBHP (0.1 mM) and different concentrations of PL.

Groups	Free TBARS	Protein-Bound TBARS
Control	0.56 ± 0.02	10.26 ± 0.20
tBHP	1.65 ± 0.03 *	14.12 ± 0.18 *
tBHP + 10 µM PL	1.46 ± 0.02 *#	12.16 ± 0.19 *#
tBHP + 25 µM PL	1.21 ± 0.03 *#	12.38 ± 0.20 *#
tBHP + 50 µM PL	1.10 ± 0.04 *#	12.10 ± 0.21 *#
tBHP + 100 µM PL	0.98 ± 0.02 *#	11.08 ± 0.20 *#

Note: * *p* < 0.05 compared to control, # *p* < 0.05 compared to tBHP.

**Table 2:** Krebs cycle enzyme activity (nmol/min/mg protein) in the brain mitochondria after treatment with tBHP (0.6 mM) and different concentrations of PL.

Groups	SDH	OGDH	Aconitase
Control	84.47 ± 0.82	4.08 ± 0.12	14.78 ± 0.20
tBHP	59.99 ± 0.83 *	3.08 ± 0.19 *	10.46 ± 0.12 *
tBHP + 50 µM PL	76.49 ± 1.38 *#	3.22 ± 0.02 *#	13.14 ± 0.09 *#
tBHP + 100 µM PL	74.03 ± 0.54 *#	3.78 ± 0.04 *#	14.23 ± 0.20 *#

Note: * *p* < 0.05 compared to control, # *p* < 0.05 compared to tBHP.

**Table 3:** The level of protein thiols (PSH, nmol/mg protein) in the brain mitochondria after treatment with tBHP (0.1 mM) and different concentrations of PL.

Groups	PSH
Control	152.75 ± 0.98
tBHP	137.86 ± 1.05 *
tBHP + 10 µM PL	138.94 ± 1.10 *
tBHP + 25 µM PL	139.28 ± 0.98 *
tBHP + 50 µM PL	144.15 ± 0.95 *#
tBHP + 100 µM PL	145.93 ± 0.96 *#

Note: * *p* < 0.05 compared to control, # *p* < 0.05 compared to tBHP.

**Table 4:** The level of GSH and GSSG (nmol/mg protein) in the mitochondrial fraction of cerebral hemispheres after treatment with FeSO_4_ (0.05 mM), succinate (5 mM), and different concentrations of PL.

Groups	GSH	GSSG	GSH/GSSG
Control	2.59 ± 0.50	0.13 ± 0.07	20.20 ± 2.05
FeSO_4_	1.63 ± 0.65 *	0.14 ± 0.08	11.96 ± 3.22 *
FeSO_4_ + succinate + 0.1 mM PL	1.69 ± 0.38 *	0.14 ± 0.09	12.09 ± 3.34 *
FeSO_4_ + succinate + 0.25 mM PL	1.55 ± 0.40 *	0.15 ± 0.08	10.58 ± 3.15 *
FeSO_4_ + succinate + 0.5 mM PL	1.89 ± 0.32 *	0.13 ± 0.07	14.15 ± 2.80 *
FeSO_4_ + succinate + 1 mM PL	2.15 ± 0.26 *#	0.14 ± 0.07	15.08 ± 3.60 *

Note: * *p* < 0.05 compared to control, # *p* < 0.05 compared to FeSO_4_.

**Table 5:** The level of GSH and GSSG (nmol/mg protein) in mitochondrial fraction of the cerebral hemispheres during the action of FeSO_4_ (0.05 mM), succinate (5 mM), PL (0.5 mM), and different concentrations of NAC.

Groups	GSH	GSSG	GSH/GSSG
Control	2.33 ± 0.12	0.17 ± 0.07	13.78 ± 1.24
FeSO_4_	1.51 ± 0.10 *	0.20 ± 0.04	7.85 ± 2.20 *
FeSO_4_ + succinate + PL	1.63 ± 0.14 *	0.21 ± 0.05	7.96 ± 2.15 *
FeSO_4_ + succinate + PL + 0.1 mM NAC	1.74 ± 0.33 *	0.23 ± 0.06	7.65 ± 2.08 *
FeSO_4_ + succinate + PL + 0.25 mM NAC	1.89 ± 0.15 *#	0.20 ± 0.07	9.50 ± 3.10 *
FeSO_4_ + succinate + PL + 0.5 mM NAC	2.04 ± 0.10 *#	0.18 ± 0.05	11.43 ± 2.05 *#
FeSO_4_ + succinate + PL + 1 mM NAC	2.43 ± 0.11 *#	0.19 ± 0.04	12.88 ± 1.93 *#

Note: * *p* < 0.05 compared to control, # *p* < 0.05 compared to FeSO_4_.

## Data Availability

The data is contained within the article.
